# St. Joseph’s—Service of Prof. Gunn

**Published:** 1875-06

**Authors:** 


### Hospitals.

### ST. JOSEPH'S HOSPITAL.

Service of Prof. Moses Gunn, M.D.

### TRAUMATIC TETANUS.

Mary Binz, Irish—married to German—æt. 34. On the 19th of February, 1875, sustained a comminuted fracture of the lower third of the tibia and fibula, with compound dislocation of the ankle joint—Potts' fracture—and was admitted to St. Joseph's Hospital, February 25th.

March 2, at 8.00 P.M., complained to the Sister in charge, of sore throat and difficulty of swallowing. Was seen at 11.00 P. M. by Dr. Scheppers, the House Physician, who directed whisky and morphia. The first, she refused to take; of the second, she had one and a quarter grains in divided (¼ gr.) doses during the night.

March 3, 10.30 A.M. Was seen by Dr. Hay. At this time her pulse was 130; respirations, 22, easy; temperature, 99.3°; she was bathed in hot sweat, her jaws were firmly clenched, the masseteric, sterno-cleido-mastoid and trapezius muscles quite rigid and hard to the touch. She swallows with difficulty, holds out her under lip with the thumb and finger of each hand to receive wine, her pupils are slightly contracted—probably from the morphia, the conjunctivæ are injected, and the face flushed. Dr. Hay ordered whisky, fl. ℥j, to be given every hour, and came to the city—four miles—to procure some ext. of calabar bean. Some of the fluid extract, said to contain one grain of the solid extract to the drop, was furnished by Messrs. Gale & Blocki, pharmacists, No. 85 South Clark street, of their own preparation.

At 4.15 P. M., the condition of the muscles was unchanged, except that the abdominal muscles were involved. The pulse was 150; respirations, 24; and temperature, 99.8°. Dr. Hay injected one drop of the extract of the calabar bean—physostigma venenosa—diluted with ten drops of water, into the right arm. The patient asked to be raised, "to get the wind off my stomach." As she was lifted, the muscles became hard and the entire trunk rigid, so as to be elevated as one piece by the hand placed under the occiput.

She swallows the whisky with difficulty, holding out the under lip with the thumb and finger and both hands to receive it, sucking it between the interstices of her clenched teeth. She talks better, her intellect is perfectly clear, and she laughs and talks cheerfully of death, which she knows to be inevitable, saying, through her tightly closed teeth, "When I am dead you can raise me and cut me up and find out all about it," without manifesting any repugnance at the idea.

Prof. Gunn arrived—in response to a telegram from Dr. Hay—at 5.20 P. M., from which time the treatment was conducted by these gentlemen jointly. At this time there was marked relaxation in the rigidity and diminution in the hardness of the muscles, the pupils were contracted likewise, the specific effect of the physostigma—1 gr.—injected at 4.15, being thus doubly demonstrated. Pulse, 155; respirations, 24; temperature, 99.8°; the injection was repeated.

At 5.35 P. M., the muscles were softened but unequally, those of the right side more than those upon the left; the relaxation of the masseters admitting the opening of the jaws to the extent of a line. The patient was much distressed by the mucus accumulated in her mouth, by reason of her inability to swallow. The sweat was not so copious, and the respiration rather abdominal than thoracic. Dr. D. Q. Scheppers kindly volunteering to watch the case through the night, was left in charge, with instructions to repeat the injections sufficiently often to secure muscular relaxation, and the doses of whisky, beef essence and milk, as she could be induced to swallow. Dr. Scheppers' notes, taken throughout the night, are as follows :

"Eight P. M., pulse, 140 ; respirations, 22 ; temperature, 98.4° ; easy, contented. Injected one drop. 9.00 P. M., "wants to sit up and have back beaten ;" pulse, sitting up, 142; respirations, 23; temperature, 100°. Injected one drop. 10.00 P. M., pulse, 144; respirations, 22; temperature, 100.2° ; passed about f℥ij of dark urine—the first since the onset of the attack, twenty-six hours—is very restless, wants to be moved often ; complains of great pain in the foot—the fracture—when moved. 11.00 P. M., very restless, wants a drink, but refuses it when offered. Pulse, 150 ; respirations, 24 ; temperature, 100°. Fans herself. Injected one drop. 12.00, midnight. Pulse, 150 ; respirations, 24 ; temperature, 100° ; fans herself ; is very easy ; calls for water continually, says "the cramps are coming up on me." Gave the "Sister" instructions about disposition of her clothing after her death. 12.30 A. M. Perspires ; wants to be raised ; is very rigid ; refuses to take whisky, persistently ; "wants the doctor to go to bed," for she will take nothing more and will not allow him to touch her. Pulse, 144 ; respirations, 24 ; temperature, 100° ; rests a little. 1.15 A. M. Perspires, refuses to take anything. Pulse, 144; respirations, 24; temperature, 100°; rests a little. 2.30 A. M. Perspires ; pulse, 142 ; respirations, 24; rests a little ; feels very hot. 3.30 A. M. Pulse, 128 ; respirations, 24 ; sleeps, resting quietly, then wakes groaning with a spasm—says "it comes from my stomach to my head ;" at intervals of from five to ten minutes wants to be fanned ; "can't swallow." 5 o'clock, A. M. Injected one drop ; pulse, 140. 7.30 A. M. Pulse, 132 ; respirations, 24. 8.15 A. M. Injected one drop. 9.15 A. M. Pulse, 138 ; respirations, 24 ; temperature, 100°. Injected one drop."

March 4, 10.00 A. M. Drs. Gunn and Hay arrived, found patient much changed for the worse ; her countenance has assumed the characteristic anxious and aged look, is much flushed and the forehead wrinkled; reflex irritability is greatly exaggerated; the jaws are tightly closed. She has still voluntary control over the facial muscles, except those supplied with innervation from the motor division of the fifth nerve. She complains of great thirst, but the application of a wet rag to her lips excites spasm, as does also touching the bed, and of "pain rising from the stomach to the throat;" is sweating profusely. The spasms extend from the diaphragm upward, and as far down the upper extremities as the elbows, the forearms and hands being exempt. Pulse, 140; respirations, 32; temperature, 100.8°. Two drops of the physostigma extract were injected into the arm, and instructions given to repeat a similar dose hourly. Her intellect was perfectly clear, and her mind composed.

Dr. Scheppers' report from this hour is as follows:

"11.00 A. M. Injected two drops of the extract. 12.00 M. A spasm occurred, which continued about a minute; she became purple in the face, was very rigid, and was thought to be dying. At 1.00 P. M. two drops were injected. At 1.30 P. M. her pulse was 160—"too fast to be counted;" respirations, 30; temperature, 101°. She "wished she could have a good drink of water." 2.00 P. M. Pulse, 158; respirations, 30; temperature, 101°. Calls for water, which she cannot swallow. Injected two drops. At 3.30 P. M. injected two drops; pulse 160; respirations, 28; temperature, 101°. Takes a few drops of wine. The injections "hurt her very little;" she "can hardly feel the puncture of the syringe." Sat up twenty minutes. 4.30 P. M. Pulse, 148; respirations, 38—during spasm, 64; injected two and a half drops. 5.30 P. M. Violent spasm; face and upper extremities purple. Five minutes after the subsidence of the spasm the pulse was 162; respirations, 44; resting quietly. Injected two drops."

At 6.30 P. M., Dr. Hay arriving, found the patient much worse. Her appearance was now truly frightful; the face was almost scarlet in color, covered with wrinkles, more especially upon the forehead, as if she had become twenty years older within six hours. She was bathed in hot sweat; her lips were livid, shrunken and dry; her teeth firmly clenched; her hands and forearms very cold and wet, contrasting strongly with the intense heat of the face and trunk. The pulse was 116, thread-like and irregular; the respirations 40, largely abdominal; the temperature, 102°. Three drops of the calabar bean extract were injected. The spasms recurred now at intervals of about two minutes, four or five in succession, of about five seconds duration each. The muscular rigidity was diminished, the muscles being quite soft in the intervals of the spasms. At 6.50 P. M., the pupils contracted unequally. At 7.40 P. M., four drops of the extract were injected, at which time her pulse was 140, irregular; respirations, 40; temperature, 101.7°; her mind perfectly tranquil and demeanor composed, except when interrupted by the prolonged cry which accompanied each spasm. The abdomen was largely distended. She describes the pains as coming up from the pubes—which she indicates with her hands—to her throat. Asked if she could live through the night, thinks "she cannot stand the pain," but is "perfectly reconciled to die."

From 9.00 P. M., the "Sister" in charge kept notes of the case as follows:

"10.40 P. M. Pulse very weak and slow, 20; temperature, 90°. 11.40 P. M. No pulse; no injection since 9.40 P. M.; very quiet, cold and clammy. 12.02 A. M. Died, in the effort to make the sign of the cross upon her breast."

March 5, 6.00 A.M. Dr. Scheppers reports the temperature, 90°. At 11.00 A. M. the temperature in the vagina was 94°, verified by Drs. Hay, Scheppers and Paul Simon.

In consequence of the violent opposition of the husband, no autopsy could be made.

March 6th. Through the kindness of Dr. Fernand Henrotin, the efficient County Physician, a Coroner's inquest was ordered in the interests of science, and the brain of the subject sent to Dr. Hay.

### NOTES BY DR. HAY.

Thirty-one grains of the calabar bean extract injected. Duration of attack, from 8.00 P. M., March 2, to 12.02 A. M., March 5—52 hours.

No muscular relaxation immediately after death. The first spasm was the beginning of the rigor mortis.

Temperature, high throughout, sank at death, and rose four degrees ten hours after death.

Nerves involved were the motor division of the fifth, the eighth, and the cervico-dorsal portion of the spinal cord.

Respiration very slightly (relatively) accelerated throughout the attack.

Profuse sweat ; no urine.

Cutaneous anæsthesia ; exalted reflex irritability ; brachial plexus involved to the elbow, not below ; roots and trunks, not peripheral branches.

Sciatic plexus, through which the original impression must have been conveyed to the centre, received no morbid impulse (efferent). Brain has how been forty-five days in strong alcohol.

WALTER HAY.

### NOTE BY PROF. GUNN.

The limb being put up in pasteboards. On 26th Dr. Gunn saw and re-dressed the fracture, using an outside splint, with a foot-piece, correcting a somewhat imperfect position of foot, and leaving the wound accessible, which latter was dressed loose, compresses saturated with a mild solution of carbolic acid. There was moderate swelling, heat and pain in the parts.

On March 1st, the wound was suppurating fairly, and the patient expressed herself as being entirely comfortable. Her cheerfulness was remarkable, considering the gravity of the injury.

